# O020. Dysfunctional analgesic mechanisms in migraine patients with ictal cutaneous allodynia

**DOI:** 10.1186/1129-2377-16-S1-A157

**Published:** 2015-09-28

**Authors:** Antonio Russo, Fabrizio Esposito, Francesca Conte, Laura Marcuccio, Michele Fratello, Giuseppina Caiazzo, Alfonso Giordano, Renata Conforti, Alessandro Tessitore, Gioacchino Tedeschi

**Affiliations:** Department of Medical, Surgical, Neurological, Metabolic and Aging Sciences, Second University of Naples, Naples, Italy; MRI Research Center SUN-FISM, Second University of Naples, Naples, Italy; Institute for Diagnosis and Care “Hermitage Capodimonte”, Naples, Italy; Department of Medicine and Surgery, University of Salerno, Baronissi, SA Italy; Neuroradiology Unit, Department of Clinical and Experimental Medicine and Surgery, Second University of Naples, Naples, Italy

## Background

Approximately two thirds of migraine patients complain of cutaneous allodynia (CA) which is defined as a pain perception evoked by ordinary non-nociceptive skin stimulation in cephalic regions during migraine attacks. CA may be underlied by the sensitization of second-order trigeminovascular neurons, belonging to the trigeminal-thalamo-cortical pathway. In this context, a crucial role seems to be played by supraspinal mechanisms related to the descending pain modulatory system [1].

## Objective

To investigate the functional pattern of pain processing pathways during trigeminal heat stimulation (THS) [2] in patients with migraine without aura, experiencing ictal cutaneous allodynia (CA) (MwoA CA+).

## Methods

Using whole-brain BOLD-fMRI, functional response to THS at three different intensities (41°, 51° and 53°C) [3] was investigated in MwoA CA+ patients compared with MwoA patients without ictal CA (MwoA CA-), in interictal period, and healthy controls (HC). Voxel-based morphometry and diffusion tensor imaging were used to explore structural or microstructural changes. Secondary analyses evaluated associations between BOLD signal change and clinical features of migraine.

## Results

During moderate-noxious THS (51°C), we observed a significantly greater activation in a) the anterior cingulate cortex in MwoA CA+ patients compared to HC and b) the middle frontal gyrus in MwoA CA+ patients compared to both MwoA CA- patients and HC. Furthermore, during high-noxious THS (53°C) a significantly decreased activation in the secondary somatosensory cortices was observed in a) MwoA CA- patients compared to both MwoA CA+ patients and HC and b) MwoA CA+ patients compared to HC. There were no structural or microstructural abnormalities between the three experimental groups. During high-noxious THS (53°C), a significant negative correlation was found between BOLD signal change in SSC and VAS scores in both MwoA CA+ patients and MwoA CA- patients. Furthermore, a significant positive correlation was found between BOLD signal change in SSC and CA severity in MwoA CA+ patients.

## Conclusions

Our findings suggest that an imbalance between the inhibition and the facilitation of pain dynamics might contribute to dysfunctional analgesic mechanisms in migraine leading to ictal CA in the course of attacks in patients with MwoA CA+. This hypothesis is further corroborated by our correlation analyses revealing that the SSC functional activity, during high-noxious THS, was positively correlated with CA in MwoA CA+ patients.

Written informed consent to publish was obtained from the patient(s).

## Funding

The study has been conducted with a grant from the Italian Foundation of Headaches (Fondazione Italiana Cefalee FI.CEF.).
